# Associations of Circulating Irisin with FNDC5 Expression in Fat and Muscle in Type 1 and Type 2 Diabetic Mice

**DOI:** 10.3390/biom11020322

**Published:** 2021-02-20

**Authors:** Songling Jiang, Lingjuan Piao, Eun Bi Ma, Hunjoo Ha, Joo Young Huh

**Affiliations:** 1 Graduate School of Pharmaceutical Sciences, College of Pharmacy, Ewha Womans University, Seoul 03760, Korea; jiang91800@gmail.com (S.J.); park.ryeongyeon@gmail.com (L.P.); hha@ewha.ac.kr (H.H.); 2 Global Development Center, Daewoong Pharmaceutical Co., Ltd., Seoul 06170, Korea; 3 College of Pharmacy, Chonnam National University, Gwangju 61186, Korea; idmy852@naver.com

**Keywords:** irisin, FNDC5, diabetes, metabolism, muscle, adipose tissue

## Abstract

Irisin is an exercise-induced myokine, suggested to exert beneficial effects on metabolism. However, the studies on the regulation of irisin secretion and the expression of its precursor FNDC5 have shown conflicting data. The discrepancies among previous correlation studies in humans are related to the heterogeneity of the study population. The fact that irisin is not only a myokine but also an adipokine leads to the further complexity of the role of irisin in metabolic regulation. In this study, we examined the regulation of FNDC5 expression and irisin in circulation in both type 1 and type 2 diabetic mice, and their potential relationships with metabolic parameters. In streptozotocin (STZ)-induced type 1 diabetic mice, high-fat diet (HFD)-induced obese mice and *db/db* mice, the circulating irisin as well as FNDC5 gene expression in subcutaneous fat was downregulated. Muscle FNDC5 expression was only significantly lower in STZ mice, and epididymal fat FNDC5 expression was unaltered. It is interesting to note that plasma irisin levels correlated positively with subcutaneous fat FNDC5 expression, but not epididymal fat or muscle. Moreover, both irisin levels and subcutaneous fat FNDC5 correlated negatively with markers of insulin resistance. These results suggest a regulatory role for subcutaneous fat-derived FNDC5/irisin in metabolic disease.

## 1. Introduction

Irisin is a myokine, identified as a proteolytic cleavage product of fibronectin type III domain-containing protein 5 (FNDC5), a transmembrane protein expressed mainly in skeletal muscle [1]. Irisin is known to be dependent on proliferator-activated receptor gamma coactivator 1-alpha (PGC1α), a major mediator of the effect of exercise in muscle [2]. In the original report by Bostrom et al., PGC1α overexpression led to the increased expression of FNDC5, which is then secreted as irisin into circulation by exercise [1]. The discovery of irisin has highlighted the vital role of crosstalk between muscle and other tissues, such as adipose tissue, liver, and bone, in the regulation of metabolism [3]. It has been proposed that irisin mediates the beneficial effects of exercise on metabolism by upregulation of uncoupling protein 1 (UCP1) expression and the subsequent elevation of energy expenditure, resulting in the amelioration of obesity-induced insulin resistance [4,5]. Since the first report in 2012, the therapeutic effects of irisin on metabolism have been evaluated in many reports, most of which were interventional animal studies. In high-fat diet (HFD)-induced diabetic mice, irisin treatment reduced epididymal fat mass and improved glucose tolerance as well as lipid profiles in serum, compared to control mice [6]. In another study, FNDC5/irisin ameliorated glucose and lipid metabolism by enhancing lipolysis via the cAMP-PKA-HSL/perilipin pathway and suppressing adipogenesis via the activation of Wnt signaling [7,8]. Furthermore, the subcutaneous perfusion of irisin improved insulin sensitivity in the liver [9], a finding which was further confirmed by the same investigators using FNDC5 knockout mice. In those animals, FNDC5 deficiency aggravated HFD-induced hepatic lipid accumulation, while FNDC5 overexpression alleviated hepatosteatosis through AMPK/mTOR-mediated autophagy restoration [10]. It is interesting to note that the anti-diabetic activity of irisin was also reported in insulin-deficient type 1 diabetic mice. Duan et al. observed that recombinant irisin treatment in streptozotocin (STZ)-induced diabetic mice significantly lowered blood glucose levels, partly by improving the expression of metabolic genes in the skeletal muscle and adipose tissue [11]. Taken together, these data indicate that irisin is a promising therapeutic candidate for the prevention and treatment of both type 1 and type 2 diabetes.

In contrast to the growing evidence with respect to the therapeutic potential of irisin, studies on the regulation of irisin and its putative role in metabolic diseases in humans have shown conflicting data. Controversy exists primarily with regard to the association between circulating irisin and body mass index (BMI). Whereas some studies have shown that irisin is positively correlated with BMI [12,13,14,15,16], others have reported the opposite relationship [17,18]. Similar divergent results have been reported for the correlation between circulating irisin and insulin resistance [19]. Although most studies agree that circulating irisin is positively correlated with insulin resistance, no consensus opinion exists regarding the association of irisin and metabolic syndrome [15,16,20,21,22,23,24,25,26,27,28]. Those who observed that a reduction in irisin is associated with an increased risk of hyperglycemia or metabolic syndrome suggest the protective role of irisin against insulin resistance, similar to the relationship observed with adiponectin in metabolic diseases. Conversely, those that have shown increased irisin levels in metabolic syndrome point to the compensatory role of irisin or even irisin-resistance, a phenomenon that resembles insulin. Relatively consistent results were observed in populations with type 2 diabetes (T2DM). In most clinical studies, including meta-analyses, lower levels of irisin in patients with T2DM compared to controls have been reported [19,24,29]. However, data regarding irisin in patients with type 1 diabetes (T1DM) are lacking.

The complexity of irisin’s role in metabolic regulation also derives from the fact that irisin is not only a myokine but also an adipokine. Muscle is known to be a dominant organ for the expression and secretion of irisin, but we and others have shown that adipocytes are also responsible for the expression of FNDC5 and the secretion of irisin [8,17,30]. In healthy individuals, most of the irisin in circulation derives from muscle cells, which is induced in response to exercise [30]. However, studies have suggested that although lower in relative expression compared to muscle, adipose tissue FNDC5 expression significantly correlates with the circulating irisin levels, especially in metabolically dysregulated states, which further supports the hypothesis that irisin may play a compensatory role as an adipokine secreted in response to altered metabolic state [17]. However, the discordance of the data on the direction of correlation makes it difficult to draw firm conclusions [30,31,32,33,34]. The contrasting role of distinct adipose tissue depots [35], including subcutaneous and visceral fat, in metabolism could also be implicated in the inconsistent findings.

The discrepancies among previous results from human subjects are related to confounding variables such as age, sex, race, and physical status. The fact that most patients with metabolic disease are under pharmacotherapy further contributes to the heterogeneity of the study population. In addition, the conflicting results in patients with metabolic syndrome may be associated with differences in the severity or duration of insulin resistance. Therefore, a well-controlled study is needed to verify the relationship between irisin secretion, FNDC5 expression, and diabetes. In this study, we have explored the regulation of FNDC5 expression and plasma irisin concentration in three different diabetic models: (i) STZ-induced type 1 diabetic mice, (ii) HFD-induced type 2 diabetic mice, and (iii) genetically obese *db/db* mice. In each group, the circulating irisin levels and tissue expression of FNDC5 in skeletal muscle, epididymal fat and subcutaneous fat were measured. The potential relationships between plasma irisin, the tissue expression of FNDC5, and metabolic parameters, such as insulin sensitivity, were investigated to gain a better understanding of the role of irisin in the pathophysiology of metabolic disease.

## 2. Materials and Methods

### 2.1. Animals

Male C57BL/6J, *db/m*, and *db/db* mice (Japan SLC Inc., Hamamatsu, Japan) were housed at 22 ± 2 °C, 50–60% humidity, with 12 h light/12 h dark cycles in a pathogen-free room. Water and food were provided ad libitum. Urine samples were collected from mice housed in metabolic cages for 24 h.

#### 2.1.1. Streptozotocin-Induced Type 1 Diabetic Mice

Experimental diabetes was induced in six-week-old mice by intraperitoneal injection of 50 mg/kg STZ for 5 days [36]. STZ was dissolved in 0.1 M sodium citrate buffer solution and kept on ice until ready for injection (pH 4.5). Age-matched controls were administered with an equivalent volume of vehicle (*n* = 11 for each group). All mice were euthanized at 12 weeks post-STZ or vehicle injection. 

#### 2.1.2. High-Fat Diet-Induced Obese Mice

Eight-week-old male mice were used in this study. Mice were fed with either normal diet (ND, D12450, 10 kcal% fat, Research Diets, Inc. New Brunswick, NJ, USA) or HFD (D12492, 60 kcal% fat, Research Diets, Inc.) for 12 weeks (*n* = 7 for each group) [37]. 

#### 2.1.3. *db/m* and *db/db* Mice

Six-week-old *db/m* (*n* = 8) and *db/db* (*n* = 9) mice were fed a standard chow diet (LabDiet 5053, PMI Nutrition International, St. Louis, MO, USA) and sacrificed at 20 weeks old. 

### 2.2. Blood Glucose, Lipid, and Hormonal Analysis

Blood samples were collected using heparinized syringes before euthanasia and were centrifuged at 3000 rpm for 20 min at 4 °C to collect plasma. Blood glucose was measured by glucometer (OneTouch Ultra, Milpitas, CA, USA). Blood hemoglobin A1c (HbA1c) level was measured using the DCA2000 HbA1c reagent kit (SIEMENS Healthcare Diagnostics, Inc., Tarrytown, NY, USA). Plasma insulin was measured using a commercial ELISA kit (EZRMI-13K, EMD Millipore Corporation, Billerica, MA, USA). Plasma free fatty acid (EFFA-100, BioAssay Systems, Hayward, CA, USA), plasma triglyceride (ETGA-200, BioAssay Systems), plasma total cholesterol (ECCH-100, BioAssay Systems), and plasma low-density lipoprotein cholesterol/very low-density lipoprotein (LDL/VLDL) cholesterol and high-density lipoprotein (HDL) cholesterol were measured using assay kits (E2HL-100, BioAssay Systems). Plasma lipid peroxide (LPO) level was measured by the thiobarbituric acid method. Plasma irisin was measured using commercially available ELISA kits (Phoenix Pharmaceuticals, Burlingame, CA, USA), according to the manufacturer’s instruction. Glucose tolerance test (GTT) was performed after 16 h fasting. Blood glucose levels were measured at 0, 15, 30, 60, 90, and 120 min after intraperitoneal injection of 1.0 g/kg body weight glucose. Collected urine samples were centrifuged at 3000 rpm for 20 min at 4 °C and then the volume was measured.

### 2.3. Organ Collection

After collecting blood, the mice were perfused with 1× PBS (pH 7.4). Subcutaneous fat, epididymal fat, brown fat, kidney, and muscle were harvested and kept on cold PBS, and then the weight was measured. All tissues were stored at −80 °C until further analysis.

### 2.4. Gene Expression Analysis by Quantitative PCR

Total RNA was extracted from quadriceps muscle, subcutaneous and epididymal adipose tissue using Trizol (Invitrogen, Carlsbad, CA, USA) according to standard protocols [38]. Expression of mRNA was measured by real-time PCR using Rotor-Gene Q (QIAGEN, Hilden, Germany) with 20 μL reaction volume consisting of cDNA transcripts, primer pairs, and SYBR Green PCR Master Mix (Enzynomics, Daejeon, Korea). Quantifications were normalized to 18S in each reaction.

### 2.5. Statistical Analysis

SPSS was used for the statistical analyses. Differences between groups were compared by ANOVA with subsequent Fisher’s significant difference method. The associations of circulating irisin levels and FNDC5 expression in muscle and fat with various parameters were calculated with Pearson’s correlation coefficients. Data are presented as mean ± SEM with *p* value of < 0.05 as the criterion for a statistically significant difference.

## 3. Results

### 3.1. FNDC5 Expression and Circulating Irisin Levels in Streptozotocin-Induced Type 1 Diabetic Mice

We first examined the levels of circulating irisin and tissue FNDC5 expression in type 1 diabetic mice. STZ-induced diabetic mice were lower in terms of body weight compared to control mice (Table 1). The metabolic parameters showed that STZ mice had significantly higher blood glucose, blood HbA1c, free fatty acid, triglyceride, and LDL/VLDL cholesterol levels compared to controls. In addition, kidney weight and urine volume per day were significantly elevated in STZ mice.

**Figure 1:**
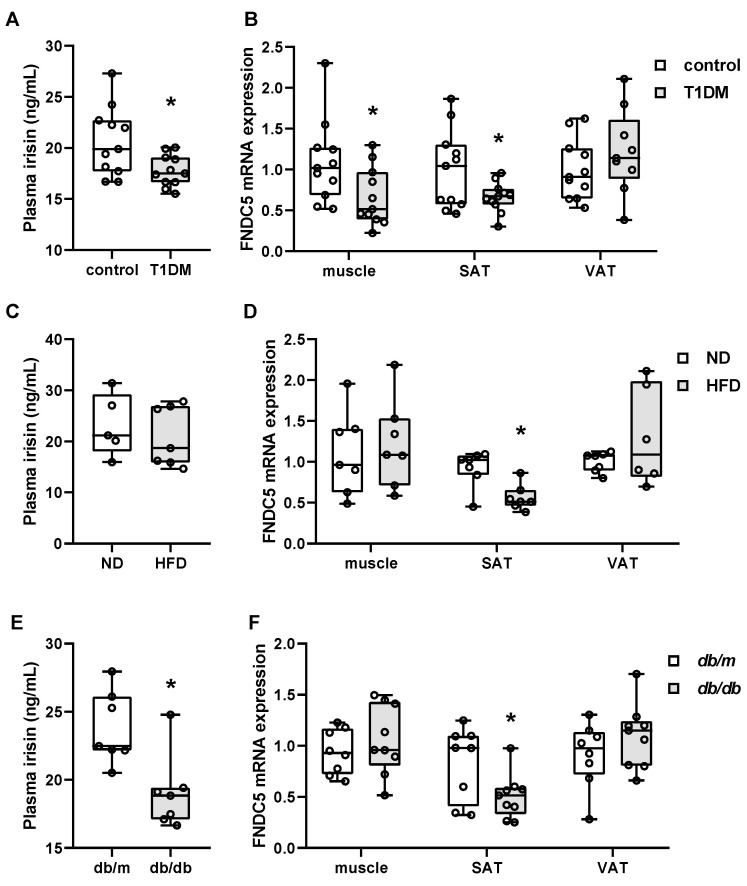
Circulating irisin and tissue FNDC5 gene expression in type 1 and type 2 diabetic mice. (**A**,**C**,**E**) Plasma irisin was measured in streptozotocin-induced type 1 diabetic (T1DM) mice (*n* = 11/group), high-fat diet (HFD)-induced obese mice (*n* = 7/group), and genetically obese *db/db* mice (*n* = 8 for *db/m*, *n* = 9 for *db/db*). (**B**,**D**,**F**) Muscle, subcutaneous adipose tissue (SAT) and visceral adipose tissue (VAT) FNDC5 gene expression was measured by real-time PCR. * *p* < 0.05 compared to the control, normal diet (ND), and *db/m*, respectively.

There are only limited reports wherein the circulating irisin and tissue expression of FNDC5 were both measured in the same experimental design [17,30,34]. Here, we have examined the plasma level of irisin, as well as the FNDC5 gene expression in muscle, subcutaneous fat, and epididymal fat. As shown in Figure 1A, plasma irisin levels were significantly lower in STZ mice compared to the control. In line with the circulating levels of irisin, muscle and subcutaneous FNDC5 mRNA expression were also downregulated in STZ mice (Figure 1B). However, there was no change in epididymal fat FNDC5 gene expression.

**Figure 2:**
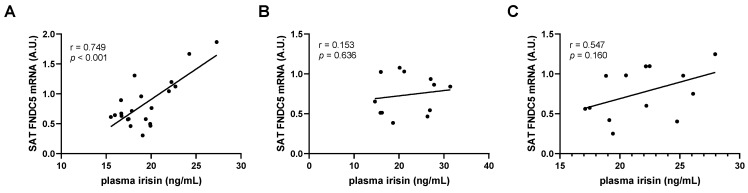
Association of circulating irisin and subcutaneous adipose tissue FNDC5 gene expression in type 1 and type 2 diabetic mice. Pearson’s correlation coefficients of plasma irisin with subcutaneous adipose tissue (SAT) FNDC5 mRNA levels in (**A**) streptozotocin-induced type 1 diabetic and control mice, (**B**) high-fat diet-induced obese and normal diet-fed mice, and (**C**) genetically obese *db/db* mice and *db/m* controls. A.U. = arbitrary units.

Next, to evaluate the correlation between circulating irisin, FNDC5 expression, and metabolic biomarkers in STZ-induced diabetic mice, we performed bivariate regression analysis (Table 2). Correlations of plasma irisin levels with metabolic parameters revealed that circulating irisin exhibited a positive association with body weight. In contrast, blood glucose, HbA1c, and urine volume were negatively associated with irisin levels. Free fatty acids also had a negative association, although only marginally significantly (*p* = 0.054). Triglycerides and total cholesterol levels were not associated with circulating irisin. Interestingly, the circulating irisin levels had a strong positive correlation with subcutaneous fat FNDC5 mRNA expression, while no association was observed with either muscle or epididymal fat expression (Table 2, Figure 2A). This correlation between circulating irisin and subcutaneous fat FNDC5 expression was reflected in the similar association pattern between subcutaneous fat FNDC5 expression and metabolic parameters. Subcutaneous fat expression was positively correlated with body weight and negatively correlated with blood glucose and urine volume. Association with HbA1c and triglyceride also had a negative trend, although this was not significant. Despite the lack of association with circulating irisin levels, muscle FNDC5 mRNA expression also showed a positive correlation with body weight and negative correlations with HbA1c, triglyceride, and LDL/VLDL cholesterol levels. It is interesting to note that epididymal FNDC5 mRNA levels were not associated with any of the parameters observed.

### 3.2. FNDC5 Expression and Circulating Irisin Levels in High-Fat Diet-Induced Obese Mice

Reports on the association of irisin with obesity and metabolic syndrome are quite controversial [15,26,27,28]. We therefore chose to examine two different animal models for obesity: HFD-induced obese mice and genetically obese *db/db* mice. In HFD mice, significant increases in body weight were observed compared to mice fed a ND. Metabolic parameters showed that HFD mice had significantly higher blood glucose, insulin, HbA1c, HOMA-IR, area under the curve (AUC) for GTT, total cholesterol, HDL cholesterol, and plasma LPO levels compared to control (Table 1).

As with the STZ model, we examined the plasma levels of irisin as well as FNDC5 gene expression in muscle, subcutaneous fat, and epididymal fat. As shown in Figure 1C, the plasma irisin levels were not significantly different between HFD and ND mice, although a trend toward downregulation may exist. When FNDC5 mRNA expression was measured in tissues, subcutaneous fat expression was the only one that was significantly regulated. While no change was observed for muscle and epididymal fat FNDC5 expression, subcutaneous fat expression was significantly downregulated in HFD mice compared to ND mice (Figure 1D).

Consistent with the above results, correlation analyses showed that subcutaneous fat FNDC5 mRNA expression was, but plasma irisin and muscle and epididymal fat FNDC5 expression were not, associated with the metabolic parameters (Table 3). Subcutaneous fat FNDC5 expression was negatively correlated with body weight, GTT AUC, total cholesterol, HDL cholesterol, and plasma LPO levels, whereas free fatty acid levels were positively correlated. Glucose, insulin, and HOMA-IR values were also negatively correlated, although they were marginally significant. The lack of change in circulating irisin levels led to no associations with muscle or fat FNDC5 expression (Table 3, Figure 2B). It is interesting to note that epididymal fat FNDC5 mRNA levels had a negative association trend with subcutaneous fat expression (*r* = −0.534, *p* = 0.074).

### 3.3. FNDC5 Expression and Circulating Irisin Levels in db/m and db/db Mice

We next examined the circulating irisin and FNDC5 expression levels in *db/db* mice. Twenty-week-old *db/db* mice had significantly increased body weight and fat weight in various depots (subcutaneous, epididymal, and brown fat) compared to non-diabetic *db/m* controls. Evident insulin resistance was observed in *db/db* mice with significantly higher blood glucose and HbA1c levels (Table 1).

Consistent with the other animal models used in this study, plasma irisin levels were significantly lower in *db/db* mice compared to *db/m* mice (Figure 1E). In line with the circulating levels of irisin, subcutaneous FNDC5 expression was also downregulated (Figure 1F). However, there was no change in muscle or epididymal fat FNDC5 gene expression. 

In the same context as the results from the STZ-induced diabetic mice, the circulating irisin levels had a marginal positive correlation with subcutaneous fat FNDC5 expression (Figure 2C), in contrast to no association with either muscle or epididymal fat FNDC5 expression. Consequently, the results of the correlation analyses showed the same pattern between plasma irisin and subcutaneous fat FNDC5 expression in terms of metabolic parameters. Correlation with metabolic parameters revealed that circulating irisin and subcutaneous FNDC5 mRNA expression had negative associations with body weight, as well as subcutaneous, epididymal, and brown fat weight (Table 4). Blood glucose, HbA1c, GTT AUC, and urine volume had negative correlations with both plasma irisin and subcutaneous fat FNDC5 expression, but the significance was stronger for subcutaneous fat FNDC5 expression. Consistent with the results from HFD-induced obese mice, epididymal FNDC5 mRNA levels were negatively correlated with subcutaneous fat FNDC5 expression.

## 4. Discussion

In this study, we have examined the relevance of circulating irisin and tissue FNDC5 expression to glucose and lipid metabolism. In particular, the question of which tissue contributes more to circulating irisin and metabolic regulation in diabetes has been delineated through correlation analysis. The highlights include (i) the consistent downregulation of circulating irisin in both T1DM and T2DM; (ii) the positive relationship between circulating irisin and subcutaneous fat FNDC5 expression, but not epididymal fat or muscle in T1DM; (iii) the negative correlation between subcutaneous fat FNDC5 expression and metabolic parameters, such as blood glucose and lipid levels, in both T1DM and T2DM. Together, the results suggest a regulatory role for subcutaneous fat-derived FNDC5/irisin in metabolism. Here, well-designed animal models with consistent results add value to previous controversial reports.

Irisin was primarily discovered as an exercise-induced myokine. We and others have confirmed that in healthy human individuals, muscle mass is an independent predictor of circulating irisin [12,13], which may be important in light of the well-characterized phenomenon of age-related muscle atrophy [39]. Although there are conflicting results on changes in FNDC5 expression in muscles after exercise, due to the high degree of heterogeneity between study designs [31,34,40,41], the strong correlation between muscle FNDC5 expression and muscle mass implies that the circulating irisin in response to exercise is attributable to FNDC5 expression in muscle. In metabolic diseases, however, higher levels of complexity exist due to diverse factors that contribute to the pathophysiology, as well as the participation of various metabolic tissues, including adipose tissue and liver, which makes generalizable conclusions difficult with regard to how irisin/FNDC5 is regulated. To date, there are limited reports on the regulation of FNDC5 expression and/or irisin production in metabolic disease. In particular, the lack of measurement of circulating irisin and muscle, as well as fat FNDC5 expression collectively, has led to difficulties in arriving at solid conclusions. Here, we show consistent results among animal models, where circulating irisin levels are decreased along with subcutaneous fat FNDC5 mRNA expression in both T1DM and T2DM.

In 2013, two different groups first reported the evidence for irisin as an adipokine [17,30]. Roca-Rivada et al. [30] used rat adipose tissue explant secretomes to prove that subcutaneous and visceral fat express and secrete FNDC5. Under basal conditions, soleus muscle secreted 20% more FNDC5 than subcutaneous fat, and approximately 60% more than visceral fat, which confirms the predominant role of subcutaneous fat over visceral fat in FNDC5 production. Moreno-Navarrete et al. reported that subcutaneous and visceral adipose FNDC5 gene expression significantly decreased in association with obesity in humans [17]. Moreover, a significant association between circulating irisin and FNDC5 gene expression in adipose tissue was observed, which is in line with our data. Similar findings have also been reported in a recent human study [34]. In contrast, Kazeminasab et al. found that both protein and mRNA levels of muscle FNDC5 were higher in HFD mice compared to controls, whereas no significant difference was observed in adipose tissue FNDC5 expression [31]. They did not observe any relationship between FNDC5 mRNA and circulating irisin, suggesting that the regulation of irisin in blood does not depend on the transcriptional regulation of FNDC5 in muscle. Another study reported that FNDC5 protein expression was higher in HFD mice in both skeletal muscle and epididymal adipose tissue compared to control, and the high expression was reversed by exercise [33]. These differences among experiments could in part be explained by the adipose depot upon which the measurement was done. The two major adipose tissue depots include visceral and subcutaneous compartments. It has been clearly shown in recent years that these depots differ in phenotypic, physiological and functional characteristics [42,43]. Although increases in both are associated with metabolic risk, visceral adipose tissue is more strongly correlated with the metabolic syndrome than subcutaneous adipose tissue [44,45]. In addition, it has been observed that subcutaneous fat has a higher propensity for expression of UCP1 and browning compared to visceral fat [46]. Along the same lines, it was confirmed that irisin activates subcutaneous beige fat cells in rodents [46]. In addition to being the primary target of irisin, our data provide evidence that subcutaneous fat is also the most sensitive site in terms of the transcriptional regulation of FNDC5. Although the change in mRNA expression shown in this study does not take into account the amount of fat, it was still positively correlated with circulating irisin levels. Moreover, the circulating irisin levels and subcutaneous fat FNDC5 expression were downregulated in both type 1 and type 2 diabetes models. Therefore, the body weight was positively correlated in STZ mice, whereas it was negatively correlated in HFD and *db/db* mice, suggesting that the regulation of FNDC5 expression in fat and subsequent irisin secretion is independent of the total amount of fat, but is more related to the quality of the adipocytes. In all three animal models, negative correlations between subcutaneous fat FNDC5 expression and metabolic markers (blood glucose, HbA1c, etc.) were observed, which implies that decreased irisin levels reflect metabolic impairment. This stands in contrast to studies that observed increased circulating irisin levels, which led to the suggestion that increased fat mass leads to compensatory secretion [30]. Interestingly, although not significant, the FNDC5 levels in visceral fat tended to increase in HFD compared to ND mice. Moreover, a negative correlation between visceral and subcutaneous fat FNDC5 expression was observed in all three animal models, supporting the contrasting roles of the two adipose depots in irisin regulation. Notably, the lack of significance in plasma irisin and correlation analyses in HFD mice seems to be the result of only a small change in body weight compared to *db/db* mice, which may become more significant with a longer duration of HFD. 

Human studies have shown inconsistent results regarding irisin levels in type 1 diabetic patients. However, our results, and the only other report on STZ-induced diabetic mice, are consistent in the finding that circulating irisin as well as muscle and adipose tissue FNDC5 mRNA expression are significantly lower than controls. In this report, it was not specified in which adipose tissue depot the expression was measured [11]. Our study has shown that subcutaneous but not visceral fat FNDC5 expression is decreased in T1DM, which positively correlates with circulating irisin as well as metabolic parameters. In contrast to the unchanged muscle mRNA levels in HFD and *db/db* mice, STZ-induced diabetic mice had lower FNDC5 mRNA expression in muscle. However, the expression in muscle did not correlate with metabolic parameters, which may imply that muscle irisin levels are not as sensitive to metabolic changes as subcutaneous fat.

The question remains as to what factors contribute to the reduction in subcutaneous fat expression. In two separate studies, the effect of leptin on FNDC5 expression was examined. Either systemic leptin treatment in rats [47] or leptin treatment in subcutaneous adipose tissue explants from nonobese subjects [48] produced a decrease in FNDC5 gene expression in subcutaneous fat, suggesting leptin as a negative regulator of FNDC5 transcription in fat. In another study, resveratrol increased FNDC5 expression in mouse and human subcutaneous adipose tissue [32]. They further observed that a SIRT1 antagonist decreased FNDC5 expression in primary adipocytes, which confirmed SIRT1 as an essential activator of FNDC5. To date, there are only limited data on the regulation of FNDC5 expression in fat. The majority of reports have focused on how muscle expression of FNDC5 is regulated, and the consequential induction of adipocyte browning and enhancements to metabolism through the endocrine system. In our study, muscle FNDC5 expression was unrelated to plasma irisin levels in all three animal models. It seems that the role of fat is underestimated, as our results and those of others show that, especially in metabolic diseases, fat-derived irisin may predominate in the regulation of whole body metabolism. Therefore, the therapeutic goal for the treatment of metabolic disease may need to be more focused on the maintenance or enhancement of subcutaneous fat FNDC5/irisin levels.

In conclusion, we report herein that circulating irisin levels and subcutaneous fat, but not muscle, FNDC5 mRNA expression are significantly downregulated in T1DM and T2DM mice. Furthermore, the negative correlation with metabolic factors suggests a protective role of subcutaneous adipose tissue-derived irisin in the metabolism. There is a limitation in that FNDC5 protein expression as well as irisin secretion from each tissue was not observed, and this needs to be investigated in future studies, in addition to the related mechanisms in FNDC5 transcriptional regulation in fat. Irisin as an adipokine rather than a myokine may play an important role as a potential candidate in drug development in metabolic diseases.

## Figures and Tables

**Table 1:** General characteristics and biochemical profile of experimental animals.

	**Control**	**Type 1 Diabetic**
Body weight (g)	26.4 ± 0.3	22.3 ± 0.5 *
Kidney weight (g)	0.17 ± 0.01	0.21 ± 0.10 *
Glucose (mM)	11.9 ± 0.8	28.6 ± 0.7 *
HbA1c (%)	4.2 ± 0.1	8.2 ± 0.4 *
Free fatty acid (mM)	0.99 ± 0.12	1.37 ± 0.12 *
Triglyceride (mM)	0.24 ± 0.01	0.49 ± 0.06 *
Total cholesterol (mM)	2.49 ± 0.05	2.55 ± 0.15
LDL/VLDL-C (mM)	0.48 ± 0.02	0.64 ± 0.05 *
HDL-C (mM)	4.06 ± 0.12	3.88 ± 0.16
Urine volume (mL/day)	1.0 ± 0.2	18.7 ± 2.2 *
	**Normal Diet**	**High-Fat Diet**
Body weight (g)	25.6 ± 0.5	33.7 ± 1.7 *
Glucose (mM)	9.2 ± 1.6	13.2 ± 1.0 *
Insulin (ng/mL)	0.44 ± 0.03	0.63 ± 0.1 *
HbA1c (%)	3.9 ± 0.1	4.2 ± 0.1 *
HOMA-IR	5.79 ± 1.15	10.64 ± 1.64 *
GTT AUC	23,949 ± 720	38,944 ± 1048 *
Free fatty acid (mM)	1.42 ± 0.17	0.56 ± 0.06 *
Triglyceride (mM)	0.49 ± 0.06	0.74 ± 0.10
Total cholesterol (mM)	1.94 ± 0.31	3.01 ± 0.07 *
LDL-C (mM)	0.51 ± 0.09	0.61 ± 0.04
HDL-C (mM)	1.76 ± 0.32	2.97 ± 0.07 *
Plasma LPO (μM)	3.4 ± 0.9	10.0 ± 0.7 *
	***db/m***	***db/db***
Body weight (g)	27.7 ± 1.1	41.1 ± 2.5 *
Subcutaneous fat weight (g)	0.23 ± 0.03	1.32 ± 0.13 *
Epididymal fat weight (g)	0.29 ± 0.04	1.10 ± 0.12 *
Brown fat weight (g)	0.19 ± 0.03	0.73 ± 0.08 *
Kidney weight (g)	0.28 ± 0.03	0.34 ± 0.01
Glucose (mM)	11.7 ± 1.0	34.0 ± 2.3 *
HbA1c (%)	4.0 ± 0.1	9.8 ± 0.2 *
GTT AUC (mg dL^−1^ min)	28113 ± 1281	111747 ± 3919 *
Urine volume (mL/day)	0.57 ± 0.19	1.96 ± 0.33 *

LDL/VLDL-C, low-density lipoprotein cholesterol/very low-density lipoprotein cholesterol; HDL-C, high-density lipoprotein cholesterol; LDL-C, low-density lipoprotein cholesterol; HOMA-IR, homeostasis model assessment for insulin resistance; GTT AUC, glucose tolerance test area under the curve; LPO, lipid peroxide. Data are shown as mean ± SEM. * *p* < 0.05 vs. control, normal diet, *db/m* mice, respectively.

**Table 2:** Correlation between plasma irisin, FNDC5 mRNA, and metabolic parameters in streptozotocin-induced diabetic mice.

		Plasma Irisin	FNDC5 mRNA (Muscle)	FNDC5 mRNA (SAT)	FNDC5 mRNA (VAT)
Plasma irisin	*r*	1			
	*p*-value				
FNDC5 mRNA (muscle)	*r*	0.202	1		
	*p*-value	0.381			
FNDC5 mRNA (SAT)	*r*	0.749 *	0.061	1	
	*p*-value	0.000	0.787		
FNDC5 mRNA (VAT)	*r*	0.022	−0.205	−0.337	1
	*p*-value	0.928	0.401	0.146	
Body weight	*r*	0.542 *	0.527 *	0.504 *	−0.042
	*p*-value	0.009	0.012	0.014	0.859
Blood glucose	*r*	−0.489 *	−0.381	−0.467 *	0.176
	*p*-value	0.021	0.080	0.025	0.458
HbA1c	*r*	−0.450 *	−0.447 *	−0.383	0.298
	*p*-value	0.036	0.037	0.071	0.202
Free fatty acid	*r*	−0.449	−0.170	−0.425	0.079
	*p*-value	0.054	0.487	0.062	0.755
Triglyceride	*r*	−0.359	−0.547 *	−0.308	0.010
	*p*-value	0.110	0.010	0.163	0.968
Total cholesterol	*r*	−0.100	−0.351	−0.076	0.003
	*p*-value	0.667	0.119	0.738	0.989
LDL/VLDL-C	*r*	−0.322	−0.494 *	−0.317	0.114
	*p*-value	0.144	0.019	0.141	0.632
HDL-C	*r*	0.364	−0.065	0.183	−0.161
	*p*-value	0.096	0.779	0.414	0.509
Urine volume	*r*	−0.446 *	−0.510 *	−0.425 *	0.136
	*p*-value	0.038	0.015	0.043	0.567

Pearson’s correlation coefficients *r* and *p* values are shown. SAT, subcutaneous adipose tissue; VAT, visceral adipose tissue; LDL/VLDL-C, low-density lipoprotein cholesterol/very low-density lipoprotein cholesterol; HDL-C, high-density lipoprotein cholesterol. * *p* < 0.05.

**Table 3:** Correlation between plasma irisin, FNDC5 mRNA, and metabolic parameters in HFD-fed mice.

		Plasma Irisin	FNDC5 mRNA (Muscle)	FNDC5 mRNA (SAT)	FNDC5 mRNA (VAT)
Plasma irisin	*r*	1			
	*p*-value				
FNDC5 mRNA (muscle)	*r*	0.271	1		
	*p*-value	0.394			
FNDC5 mRNA (SAT)	*r*	0.153	0.110	1	
	*p*-value	0.636	0.721		
FNDC5 mRNA (VAT)	*r*	0.195	0.245	−0.534	1
	*p*-value	0.565	0.420	0.074	
Body weight	*r*	−0.461	0.026	−0.779*	0.283
	*p*-value	0.132	0.929	0.002	0.348
Fasting glucose	*r*	0.067	0.386	−0.450	0.427
	*p*-value	0.836	0.173	0.123	0.146
Fasting insulin	*r*	−0.368	0.047	−0.599	0.300
	*p*-value	0.265	0.884	0.051	0.370
HbA1c	*r*	−0.303	0.245	−0.444	0.266
	*p*-value	0.339	0.398	0.129	0.380
HOMA-IR	*r*	−0.172	0.198	−0.600	0.505
	*p*-value	0.614	0.536	0.051	0.113
GTT AUC	*r*	−0.116	0.110	−0.871 *	0.364
	*p*-value	0.720	0.708	0.000	0.222
Free fatty acid	*r*	−0.029	−0.069	0.655 *	−0.438
	*p*-value	0.929	0.815	0.015	0.135
Triglyceride	*r*	−0.447	0.096	−0.430	−0.128
	*p*-value	0.145	0.744	0.143	0.676
Total cholesterol	*r*	−0.061	0.456	−0.577 *	0.213
	*p*-value	0.850	0.101	0.039	0.484
LDL-C	*r*	0.108	0.128	−0.115	0.301
	*p*-value	0.738	0.663	0.707	0.317
HDL-C	*r*	−0.163	0.455	−0.602 *	0.185
	*p*-value	0.613	0.102	0.030	0.546
LPO	*r*	−0.120	0.475	−0.655 *	0.349
	*p*-value	0.710	0.086	0.015	0.242

Pearson’s correlation coefficients *r* and *p* values are shown. SAT, subcutaneous adipose tissue; VAT, visceral adipose tissue; HOMA-IR, homeostasis model assessment for insulin resistance; GTT AUC, glucose tolerance test area under the curve; LDL-C, low-density lipoprotein cholesterol; HDL-C, high-density lipoprotein cholesterol; LPO, lipid peroxide. * *p* < 0.05.

**Table 4:** Correlation between plasma irisin, FNDC5 mRNA, and metabolic parameters in *db/db* mice.

		Plasma irisin	FNDC5 mRNA (Muscle)	FNDC5 mRNA (SAT)	FNDC5 mRNA (VAT)
Plasma irisin	*r*	1			
	*p*-value				
FNDC5 mRNA (muscle)	*r*	−0.282	1		
	*p*-value	0.351			
FNDC5 mRNA (SAT)	*r*	0.547	−0.150	1	
	*p*-value	0.160	0.593		
FNDC5 mRNA (VAT)	*r*	−0.171	0.121	−0.538 *	1
	*p*-value	0.637	0.643	0.047	
Body weight	*r*	−0.688 *	0.067	−0.542 *	0.079
	*p*-value	0.009	0.778	0.037	0.763
SAT weight	*r*	−0.611 *	0.037	−0.619 *	0.063
	*p*-value	0.027	0.877	0.014	0.809
VAT weight	*r*	−0.848 *	0.150	−0.537 *	0.048
	*p*-value	0.000	0.529	0.039	0.854
Brown fat weight	*r*	−0.708 *	0.149	−0.587 *	0.305
	*p*-value	0.007	0.531	0.022	0.234
Kidney weight	*r*	−0.444	0.347	−0.451	−0.226
	*p*-value	0.129	0.133	0.092	0.384
Blood glucose	*r*	−0.593 *	0.371	−0.751 *	0.050
	*p*-value	0.033	0.118	0.002	0.854
HbA1c	*r*	−0.602 *	0.093	−0.727 *	0.087
	*p*-value	0.030	0.706	0.003	0.748
GTT AUC	*r*	−0.649 *	0.294	−0.730 *	0.070
	*p*-value	0.016	0.209	0.002	0.791
Urine volume	*r*	−0.259	0.345	−0.569*	−0.022
	*p*-value	0.392	0.137	0.027	0.933

Pearson’s correlation coefficients *r* and *p* values are shown. SAT, subcutaneous adipose tissue; VAT, visceral adipose tissue; GTT AUC, glucose tolerance test area under the curve. * *p* < 0.05.

## Data Availability

The data presented in this study are available in the figures and tables of this manuscript.
